# Comparative analysis of plasma BNP and NT-proBNP levels, and NT-proBNP/BNP ratio in patients with chronic kidney disease

**DOI:** 10.1038/s41440-025-02272-2

**Published:** 2025-07-01

**Authors:** Hideki Fujii, Shunsuke Goto

**Affiliations:** Division of Nephrology, Kobe University Graduate School of Medicine, Kobe, Japan

**Keywords:** B-type natriuretic peptide, Chronic kidney disease, Cardiovascular disease, N-terminal pro-B-type natriuretic peptide, The BNP/NT-proBNP ratio

## Abstract

Both B-type natriuretic peptide (BNP) and N-terminal pro-B-type natriuretic peptide (NT-proBNP) are clinically used for diagnosing and monitoring heart failure. However, their levels are influenced by several factors, and their impacts on chronic kidney disease (CKD) patients remain unclear. This study included 1036 patients who visited the Nephrology division at our hospital between 2014 and 2015. Plasma BNP, NT-proBNP levels and the BNP/NT-proBNP ratio were measured at each CKD stage, and their correlation with clinical factors were analyzed. This study included 1037 patients with stage 1 to stage 5D CKD (CKD 1-2, *n *= 114; CKD 3, *n *= 256; CKD 4, *n *= 266; CKD 5, *n *= 298; CKD 5D, *n *= 102). Levels of plasma BNP and NT-proBNP levels and the NT-proBNP/BNP ratio increased, and the correlation between BNP and NT-proBNP levels weakened with declining kidney function. Although various clinical factors were found to be significantly correlated with these parameters, multivariate analysis showed that male gender and hemoglobin, phosphate, and parathyroid hormone levels were significantly correlated with both plasma BNP and NT-proBNP levels. Notably, a higher NT-proBNP/BNP ratio was significantly associated with increased cardiovascular events in patients with CKD stages 4 and 5. As plasma BNP and NT-proBNP levels are influenced by various factors in patients with CKD, careful interpretation of these parameters is essential. In patients with advanced-stage CKD, the NT-proBNP/BNP ratio may be a useful predictor of CVD development.

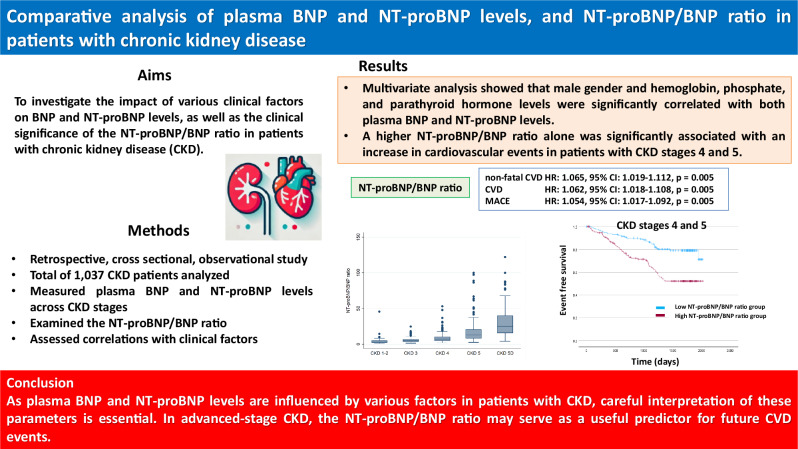

## Introduction

In patients with chronic kidney disease (CKD), cardiovascular disease (CVD) is a major complication. The incidence and mortality rates of CVD increase as kidney function declines as represented by a decreased estimated glomerular filtration rate (eGFR) [1]. Even in patients with an eGFR above 60 mL/min/1.73 m^2^, the presence of proteinuria doubles the risk of CVD mortality compared with those without proteinuria [2]. Additionally, patients with only microalbuminuria have a higher risk of both CVD incidence and mortality than those without microalbuminuria [3]. Thus, considering that CKD is a significant risk factor for CVD, increased attention to CVD in patients with CKD is necessary. Common CVDs among patients with CKD include coronary artery disease (CAD), peripheral artery disease, aortic disease, arrhythmia, and heart failure (HF). HF is reportedly the most frequent CVD in patients with CKD [4]. Therefore, preventing CVD development is essential. The relationship between CKD and CVD aligns with the recently proposed concept of the cardio-renal syndrome [5–7]. In HF, several factors, such increases levels of hormones and cytokines, decreased renal blood flow, changes in intraglomerular pressure, and alterations in intravascular blood volume, can further influence the progression of CKD [5, 7].

Thus, the presence of CKD is closely linked with the prognosis of patients with HF. However, the pathophysiological mechanisms underlying the cardiorenal association are complex and poorly understood. In managing HF, while clinical symptoms and echocardiography are essential, brain natriuretic peptide (BNP) and N-terminal pro-brain natriuretic peptide (NT-proBNP) are most commonly used biomarkers for monitoring HF. The international definition of HF, as stated by the Japanese heart failure society, the heart failure association of the European society of cardiology, and the heart failure society of America, also includes elevated levels of BNP and NT-proBNP as part of the diagnostic criteria [8]. Additionally, these societies provide a comprehensive and up-to-date perspective on natriuretic peptides for diagnosing and managing HF [9].

In patients with CKD, BNP and NT-proBNP levels are significantly influenced by several factors [10–12], making their interpretation challenging. With the advent of angiotensin receptor neprilysin inhibitor (ARNI), NT-proBNP has become more commonly used in the field of cardiology. However, because NT-proBNP levels are significantly influenced by kidney function, concerns have been raised regarding its limitations, particularly in patients with impaired kidney function. Previous studies have reported that the NT-proBNP/BNP ratio serves as an independent prognostic factor in cases of acute HF [13]. Based on this background, the present study aims to conduct a clinical investigation focusing on BNP, NT-proBNP, and the NT-proBNP/BNP ratio.

Point of view

- Clinical relevance
  NT-proBNP/BNP ratio is a strong predictor of cardiovascular outcomes in CKD patients at advanced stages.
- Future direction
  Integration of the NT-proBNP/BNP ratio into clinical practice guidelines for CVD risk assessment in CKD.
- Consideration for the Asian population
  Reflects CKD-specific pathophysiology and clinical practice in Asia, where dialysis and CKD-MBD are major considerations.

## Methods

### Study design and population

This was a retrospective observational study conducted at a single center that included patients who visited our department between December 2014 and December 2015. Among them, patients who met any of the following criteria were excluded from the present study: kidney transplant recipients, acute myocardial infarction, unstable angina pectoris, acute HF, serious infection, non-CKD, and insufficient clinical data. In addition, patients without pre-existing CKD who developed transient acute kidney injury were also excluded. Overall, 1036 patients were enrolled in the study and classified according to the CKD stage based on the eGFR and presence of proteinuria as follows; CKD stages 1 and 2 (≥60 mL/min/1.73 m^2^), stage 3 (30–59 mL/min/1.73 m^2^), stage 4 (15–29 mL/min/1.73 m^2^), stage 5 (<15 mL/min/1.73 m^2^), and stage 5D (dialysis). Collected information from the medical records included diabetes mellitus (DM), CAD, chronic HF, atrial fibrillation (AF), other CVDs, hypertension (HT), and hyperlipidemia (HL).

This study was conducted in accordance with the principles stated in the Declaration of Helsinki. Our study protocol was approved by the appropriate institutional review committee. This study is registered with the UMIN Clinical Trials Registry (UMIN-CTR) under the identifier UMIN000029758 (https://upload.umin.ac.jp/cgi-open-bin/ctr_e/ctr_view.cgi?recptno=R000033999). The ethical committee waived the need for informed consent in this study as data were retrospectively and anonymously analyzed.

### Laboratory measurements

Laboratory testing was conducted using standardized clinical laboratory methods. The remaining EDTA–2Na plasma samples were stored at −80 °C until further analysis. BNP and NT-proBNP levels were measured using the AIA-CL system (TOSOH Co., Tokyo, Japan) and the Elecsys NT-proBNP assay (Roche Diagnostics K.K., Tokyo, Japan).

### Clinical outcomes

Occurrence of CVD events and all-cause mortality in CKD stages 4 and 5 were assessed during the observational period until June 2020. The median follow-up period was 1365 days (IQR: 694–1780). Non-fatal CVD events included stable angina pectoris, acute coronary syndrome (non-fatal myocardial infarction or unstable angina pectoris), HF, aortic dissection, aortic aneurysm, and stroke. Stable angina was defined as the presence of symptoms suggestive of ischemia or electrocardiographic abnormalities, accompanied by significant coronary artery stenosis confirmed by coronary angiography. HF was defined as the presence of symptoms such as dyspnea, fatigue, pulmonary crackles and/or peripheral edema, along with elevated levels of BNP or NT-proBNP levels. CVD was defined as sudden death and death due to CAD, HF, aortic dissection, aortic aneurysm, and stroke. CVD events included CVD death and non-fatal CVD events. A major adverse cardiovascular event (MACE) was defined as the composite of all non-fatal CVD events and all-cause mortality.

### Statistical analysis

All statistical analyses were conducted using IBM SPSS statistics software version 25.0 (SPSS, Inc., Chicago, IL, USA). Continuous variables are expressed as means ± standard deviation, as well as median and interquartile range. When comparing the clinical backgrounds among the four groups, the χ-square test (for categorical variables) and one-way analysis of variance (for continuous variables) were used, followed by the Turkey’s HSD post-hoc test. The Kaplan–Meier method and log-rank test were conducted to compare the outcomes between the groups. Cox proportional hazard models were employed to adjust for confounders, including age, gender, a history of smoking, DM, a history of CVD, kidney function, and urinary protein levels. A *P* value of < 0.05 was considered statistically significant.

## Results

### Patients’ characteristics

Total 1036 patients were included in the present study and classified into CKD stages 1 and 2 (*n* = 114), stage 3 (*n* = 256), stage 4 (*n* = 266), stage 5 (*n* = 298), and stage 5D (*n* = 102). The characteristics of the patients are shown in Table 1. The mean age was 62.8 ± 16.4 years, and 663 patients (64.0%) were men. Additionally, 900 patients had HT (86.9%), and 329 patients had DM (31.8%). In patients with CKD stages 1-5, the mean eGFR and proteinuria level were 31.6 ± 23.2 and 2.0 ± 2.7, respectively. Patients with CKD stages 1 and 2 were younger, and HT was less prevalent than in patients in the other groups. As kidney function declined, HF and CAD became more prevalent, hemoglobin levels decreased, and serum phosphate and parathyroid hormone (PTH) levels increased.

**Table 1:** Patients’ characteristics

	Overall *N* = 1036	CKD 1-2 *N* = 114	CKD 3 *N* = 256	CKD 4 *N* = 266	CKD 5 *N* = 298	CKD 5D *N* = 102
Age (year)	62.8 ± 16.4	50.2 ± 18.3	60.1 ± 17.1	67.4 ± 15.8	65.5 ± 13.5	63.4 ± 12.5
Male (%)	658 (63.5)	63 (55.3)	169 (66.0)	186 (69.9)	182 (61.1)	58 (56.9)
Smoking (%)	531 (51.3)	47 (41.2)	137 (53.5)	158 (59.4)	142 (47.7)	47 (46.1)
HT (%)	900 (86.9)	69 (60.5)	220 (85.9)	242 (91.0)	276 (92.6)	93 (91.2)
DM (%)	329 (31.8)	40 (35.1)	77 (30.1)	69 (25.9)	104 (34.9)	39 (38.2)
HLp (%)	566 (54.6)	57 (50.0)	148 (57.8)	116 (43.6)	187 (62.8)	58 (56.9)
CHF (%)	95 (9.2)	5 (4.4)	15 (5.9)	41 (15.4)	23 (7.7)	11 (10.8)
CAD (%)	143 (13.8)	6 (5.3)	34 (13.3)	42 (15.8)	48 (16.1)	13 (12.7)
AF (%)	81 (7.8)	1 (0.9)	12 (4.7)	28 (10.5)	30 (10.1)	10 (9.8)
ACE-I/ARB (%)	624 (60.2)	62 (54.4)	197 (77.0)	148 (55.6)	161 (54.0)	56 (54.9)
Β-blocker (%)	337 (32.5)	20 (17.5)	84 (32.8)	92 (34.6)	96 (32.2)	45 (44.1)
Statin (%)	504 (48.6)	41 (36.0)	134 (52.3)	95 (35.7)	183 (61.4)	51 (50.0)
Hb (g/dL)	11.3 ± 1.9	13.2 ± 1.9	12.4 ± 1.6	11.2 ± 1.5	10.1 ± 1.4	10.0 ± 1.6
Alb (g/dL)	3.8 ± 0.7	3.7 ± 1.0	3.8 ± 0.8	3.9 ± 0.5	3.7 ± 0.6	3.2 ± 0.6
BUN (mg/dL)	41.2 ± 24.5^a^	14.5 ± 4.2	25.3 ± 12.4	40.5 ± 12.3	65.6 ± 23.0	N.A.
Cre (mg/dL)	2.08 (1.29-3.79)	0.74 (0.66–0.85)	1.32 (1.10–1.56)	2.24 (1.90–2.68)	5.03 (3.85–6.22)	N.A.
eGFR (mL/min/1.73m^2^)	30.4 ± 23.2^a^	78.9 ± 14.3	41.9 ± 8.9	22.2 ± 4.1	9.3 ± 3.0	N.A.
cCa (mg/dL)	9.3 ± 0.7	9.6 ± 0.5	9.5 ± 0.5	9.3 ± 0.4	8.9 ± 0.8	9.1 ± 0.8
P (mg/dL)	4.0 ± 1.1	3.4 ± 0.7	3.4 ± 0.7	3.5 ± 0.6	4.7 ± 1.0	5.1 ± 1.5
CRP (mg/dL)	0.16 (0.05–0.64)	0.09 (0.04–0.28)	0.12 (0.04–0.54)	0.20 (0.05–0.54)	0.18 (0.06–0.57)	0.59 (0.10–3.13)
U-Pro (g・gCr)^a^	0.9 (0.2–2.7)	0.3 (0.1–1.9)	0.4 (0.1–2.2)	0.5 (0.2–1.6)	2.1 (0.9–4.1)	N.A.
i-PTH (pg/mL)	111.0 (63.7–211.5)	48.0 (33.5–73.1)	70.3 (51.5–106.9)	112.8 (75.1–154.5)	250.6 (152.4–427.0)	137.2 (69.1–269.6)

*CKD* chronic kidney disease, *HT* hypertension, *DM* diabetes mellitus, *HLp* hyperlipidemia, *CHF* chronic heart failure, *CAD* coronary artery disease, *AF* atrial fibrillation, *ACE-I/ARB* angiotensin converting enzyme inhibitors/angiotensin II receptor blocker, *Hb* hemoglobin, *Alb* albumin, *Cre* creatinine, *eGFR* estimated glomerular filtration rate, *cCa* corrected calcium, *P* phosphate, *CRP* C-reactive protein, *U-Pro* urinary protein, *i-PTH* intact parathyroid hormone

^a^mean values in patients with CKD stages 1-5

### BNP levels, NT-proBNP levels, and the NT-proBNP/BNP ratio in CKD patients

**Fig. 1:**
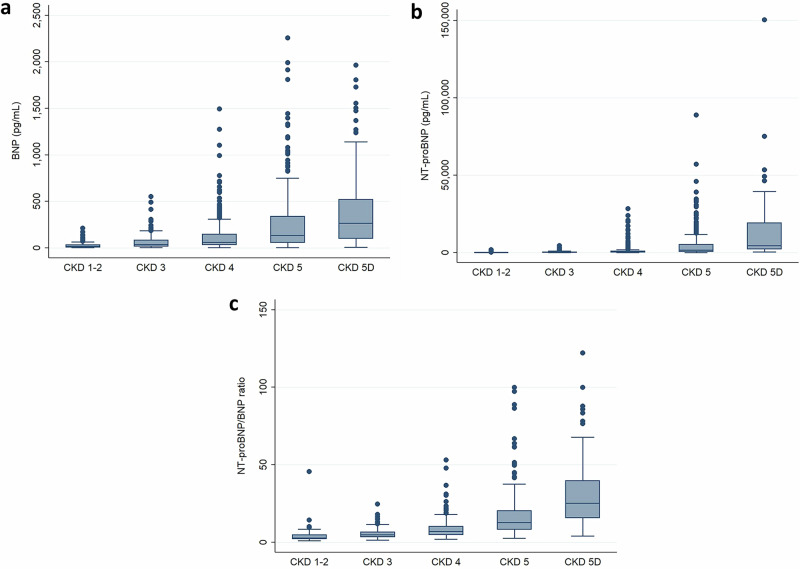
Biomarker levels in each CKD stage. **a** Plasma BNP levels in each CKD stage. CKD 1-2: 15.9 (8.3–30.7) pg/mL, CKD 3: 37.5 (15.0–83.1) pg/mL, CKD 4: 59.4 (33.2–147.4) pg/mL, CKD 5: 133.9 (58.2–338.6) pg/mL, CKD 5D: 264.3 (100.4–520.7) pg/mL. **b** Plasma NT-proBNP levels in each CKD stage. CKD 1-2: 44.5 (23.2–98.9) pg/mL, CKD 3: 165.8 (71.7–463.5) pg/mL, CKD 4: 445.4 (216.7–939.0) pg/mL, CKD 5: 1686.5 (571.4–5308.0) pg/mL, CKD 5D: 4489.5 (2242.0–19213.0) pg/mL. **c** The NT-proBNP/BNP ratio in each CKD stage. CKD 1-2: 2.9 (2.3–4.7), CKD 3: 4.8 (3.5–6.8), CKD 4: 7.2 (4.9–10.2), CKD 5: 12.7 (8.3–20.3), CKD 5D: 25.1 (15.7–39.7). CKD chronic kidney disease, BNP brain natriuretic peptide, NT-proBNP N-terminal pro-brain natriuretic peptide

**Fig. 2:**
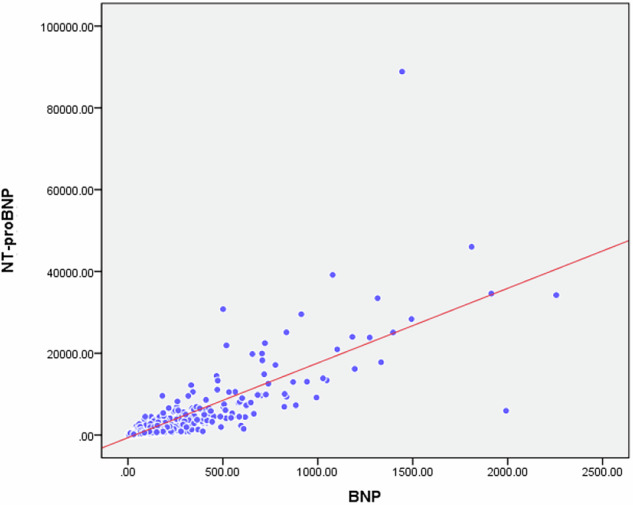
Relationship between BNP and NT-proBNP in patients with CKD stage 1-5. BNP, brain natriuretic peptide; NT-proBNP, N-terminal pro-brain natriuretic peptide; CKD, chronic kidney disease

The mean BNP and NT-proBNP levels in all the patients were 169.4 ± 278.6 pg/mL and 3,168.8 ± 8,844.8 pg/mL, respectively; excluding CKD stage 5D, they were 143.5 ± 241.5 pg/mL and 2,163.0 ± 5,985.4 pg/mL, respectively. The mean plasma BNP levels in each group were 27.8 ± 34.5 pg/mL (CKD stages 1 and 2), 64.2 ± 76.5 pg/mL (CKD stage 3), 132.2 ± 195.3 pg/mL (CKD stage 4) and 265.3 ± 343.1 pg/mL (CKD stage 5), and 406.2 ± 440.6 pg/mL (CKD stage 5D) (Fig. 1a). The mean plasma NT-proBNP levels in each group were 131.0 ± 264.6 pg/mL (CKD stage 1 and 2), 355.2 ± 512.5 pg/mL (CKD stage 3), 1459.8 ± 3552.3 pg/mL (CKD stage 4) and 5112.9 ± 9334.6 pg/mL (CKD stage 5), and 12,378.6 ± 19,383.1 pg/mL (CKD stage 5D) (Fig. 1b). Both BNP and NT-proBNP levels increased as kidney function declined, with the changes in NT-proBNP levels being remarkable. Regarding the NT-proBNP/BNP ratio, although the trend of the changes was similar to the BNP and NT-proBNP levels, it was remarkably high in CKD stage 5D (Fig. 1c). Although the correlation between BNP and NT-proBNP levels was statistically significant in CKD stages 1-5 (Fig. 2), it became weaker as CKD stage increased (CKD stages 1 and 2; *r* = 0.914, *p* < 0.0001; CKD stage 3; *r* = 0.865, *p* < 0.0001; CKD stage 4; *r* = 0.878, *p* < 0.0001; CKD stage 5; *r* = 0.786, *p* < 0.0001; CKD stage 5D; *r* = 0.691, *p* < 0.0001; CKD stage 1-5; *r* = 0.814, *p* < 0.0001). The distribution of the BNP/NT-proBNP ratio was shown in supplementary Fig. 1.

### Correlation of BNP levels, NT-proBNP levels, and the NT-proBNP/BNP ratio with clinical factors

Factors significantly related to BNP levels, NT-proBNP levels, and the NT-proBNP/BNP ratio in patients with CKD stages 1-5 are shown in Table 2. Interestingly, the number of clinical factors significantly related to the NT-proBNP/BNP ratio was less than those significantly related to BNP and NT-proBNP levels. Furthermore, in CKD stage 5D, the related factors were greatly different from those in patients not on dialysis (Table 3). Among these patients, nothing was significantly correlated with the NT-proBNP/BNP ratio. Therefore, we conducted multivariate analysis to evaluate the independent factors for BNP levels, NT-proBNP levels, and the NT-proBNP/BNP ratio in patients with CKD stages 1-5 only. Male gender, hemoglobin levels, phosphate levels, and intact PTH levels were independent factors for BNP levels, where male gender, smoking, HF, AF, use of ACE-I/ARB, hemoglobin levels, phosphate levels, and intact PTH levels were independent factors for NT-proBNP levels. Meanwhile, hemoglobin levels, eGFR, phosphate levels, intact PTH levels, and the amount of U-Pro were independent factors for the NT-proBNP/BNP ratio (Table 4). In patients with CKD stage 5D, HF and AF were independent determining factors for BNP levels, and HT and corrected Ca were independent determining factors for NT-proBNP (Table 5). Furthermore, we divided these patients into tertiles based on the BNP/NT-proBNP ratio and examined their clinical backgrounds (Supplementary Table 1). We found that in the group with a high BNP/NT-proBNP ratio, more than 95% of the patients were in CKD stages 4, 5 or 5D, suggesting that many of these patients had multiple risk factors for CVD, as indicated by the associated factor analysis.

**Table 2:** Univariate analysis of the correlation of BNP levels, NT-proBNP levels, and the NT-proBNP/BNP ratio with clinical factors in patients with CKD stages 1-5

	BNP		NT-proBNP		NT-proBNP/BNP	
	*r*	*P*	*r*	*P*	*r*	*P*
Age	0.138	<0.001	0.051	0.118	0.046	0.163
Male	0.125	<0.001	0.092	0.005	−0.006	0.846
Smoking	0.129	<0.001	0.104	0.001	0.009	0.776
HT	0.207	<0.001	0.227	<0.001	0.094	0.004
DM	-0.008	0.795	−0.008	0.795	−0.014	0.661
HLp	0.078	0.018	0.078	0.018	0.014	0.674
CHF	0.166	<0.001	0.159	<0.001	0.001	0.079
CAD	0.220	<0.001	0.195	<0.001	0.064	0.050
AF	0.250	<0.001	0.219	<0.001	−0.014	0.680
ACE-I/ARB	−0.157	<0.001	-0.146	<0.001	−0.047	0.152
β-blocker	0.211	<0.001	0.188	<0.001	0.000	0.994
Statin	0.104	0.001	0.117	0.001	0.073	0.025
Hb	−0.398	<0.001	−0.365	<0.001	−0.423	<0.001
eGFR	−0.320	<0.001	−0.280	<0.001	−0.439	<0.001
cCa	−0.100	0.002	−0.081	0.013	−0.079	0.016
P	0.355	<0.001	0.370	<0.001	0.379	<0.001
i-PTH	0.398	<0.001	0.515	<0.001	−0.385	<0.001
U-Pro	0.248	<0.001	0.334	<0.001	0.211	<0.001

*BNP* brain natriuretic peptide, *NT-proBNP* N-terminal pro brain natriuretic peptide, *HT* hypertension, *DM* diabetes mellitus, *HLp* hyperlipidemia, *CHF* chronic heart failure, *CAD* coronary artery disease, *AF* atrial fibrillation, *ACE-I/ARB* angiotensin converting enzyme inhibitors/angiotensin II receptor blocker, *Hb* hemoglobin, *eGFR* estimated glomerular filtration rate, *cCa* corrected calcium, *P* phosphate, *i-PTH* intact parathyroid hormone, *U-Pro* urinary protein

**Table 3:** Univariate analysis of the correlation of BNP levels, NT-proBNP levels, and the NT-proBNP/BNP ratio with clinical factors in patients with CKD stage 5D

	BNP		NT-proBNP		NT-proBNP/BNP	
	*r*	*P*	*r*	*P*	*r*	*P*
Age	0.222	0.025	0.040	0.686	−0.162	0.103
Male	0.041	0.681	0.067	0.504	0.036	0.716
Smoking	0.102	0.309	0.213	0.032	−0.006	0.951
HT	0.207	<0.001	−0.345	<0.001	−0.163	0.102
DM	−0.008	0.795	−0.023	0.098	0.098	0.327
HLp	0.019	0.852	−0.131	0.188	−0.054	0.589
CHF	0.336	0.001	0.068	0.499	−0.110	0.269
CAD	0.032	0.746	0.004	0.970	0.020	0.842
AF	0.369	0.001	0.106	0.290	−0.069	0.490
ACE-I/ARB	−0.039	0.700	−0.226	0.023	−0.173	0.082
β-blocker	0.018	0.857	−0.070	0.482	−0.037	0.715
Statin	−0.004	0.965	−0.149	0.134	−0.057	0.572
Hb	0.036	0.720	−0.042	0.672	−0.054	0.587
cCa	−0.266	0.007	−0.135	0.177	0.159	0.112
P	−0.029	0.836	−0.111	0.267	−0.062	0.537
i-PTH	−0.029	0.772	0.043	0.671	0.176	0.077

*BNP* brain natriuretic peptide, *NT-proBNP* N-terminal pro-brain natriuretic peptide, *HT* hypertension, *DM* diabetes mellitus, *HLp* hyperlipidemia, *CHF* chronic heart failure, *CAD* coronary artery disease, *AF* atrial fibrillation, *ACE-I/ARB* angiotensin converting enzyme inhibitors/angiotensin II receptor blocker, *Hb* hemoglobin, *cCa* corrected calcium, *P* phosphate, *i-PTH* intact parathyroid hormone

**Table 4:** Multivariate analysis of the correlation of BNP levels, NT-proBNP levels, and the NT-proBNP/BNP ratio with clinical factors in patients with CKD stages 1-5

	BNP		NT-proBNP		NT-proBNP/BNP	
	*β*	*P*	*β*	*P*	*β*	*P*
Age	0.046	0.150	-	-	-	-
Male	0.098	0.005	0.100	0.006	-	-
Smoking	0.068	0.043	0.033	0.348	-	-
HT	−0.005	0.866	−0.029	0.369	−0.027	0.369
HLp	0.072	0.205	0.004	0.944	-	-
CHF	0.074	0.016	0.028	0.383	-	-
CAD	−0.014	0.673	0.029	0.388	-	-
AF	0.138	<0.001	0.046	0.149	-	-
ACE-I/ARB	−0.062	0.044	−0.009	0.782	-	-
β-blocker	0.016	0.611	−0.028	0.413	-	-
Statin	−0.031	0.588	0.006	0.918	−0.017	0.556
Hb	−0.239	<0.001	−0.214	<0.001	−0.164	<0.001
eGFR	0.008	0.850	0.010	0.799	−0.219	<0.001
cCa	−0.022	0.444	−0.011	0.708	0.001	0.979
P	0.194	<0.001	0.248	<0.001	0.127	<0.001
i-PTH	0.158	<0.001	0.149	<0.001	0.156	<0.001
U-Pro	0.014	0.635	0.019	0.560	0.102	0.001

BNP; adjusted by age, male gender, smoking, HT, HLp, CHF, CAD, AF, ACE-I/ARB, β-blocker, statin, Hb, eGFR, cCa, P, i-PTH, and U-ProNT-proBNP; adjusted by male gender, smoking, HT, statin, Hb, eGFR, cCa, P, i-PTH, and U-ProNT-proBNP/BNP ratio; adjusted by HT, HLp, CHF, CAD, AF, ACE-I/ARB, β-blocker, statin, Hb, eGFR, cCa, P, i-PTH, and U-Pro

*BNP* brain natriuretic peptide, *NT-proBNP* N-terminal pro-brain natriuretic peptide, *HT* hypertension, *DM* diabetes mellitus, *HLp* hyperlipidemia, *CHF* chronic heart failure, *CAD* coronary artery disease, *AF* atrial fibrillation, *ACE-I/ARB* angiotensin converting enzyme inhibitors/angiotensin II receptor blocker, *Hb* hemoglobin, *eGFR* estimated glomerular filtration rate, *cCa* corrected calcium, *P* phosphate, *i-PTH* intact parathyroid hormone; U-Pro, urinary protein

**Table 5:** Multivariate analysis of the correlation of BNP and NT-proBNP levels with clinical factors in patients with CKD stage 5D

	BNP		NT-proBNP	
	*Β*	*P*	*β*	*P*
Age	0.138	0.128	-	-
Smoking	-	-	0.184	0.051
HT	-	-	−0.299	0.003
HF	0.203	0.037	-	-
AF	0.254	0.011	-	-
ACE-I/ARB	-	-	−0.103	0.300
cCa	−0.258	0.004	-	-

BNP; adjusted by age, HF, AF, and cCaNT-proBNP; adjusted by smoking and ACE-I/ARB

*BNP* brain natriuretic peptide, *NT-proBNP* N-terminal pro-brain natriuretic peptide, *HT* hypertension, *HF* heart failure, *AF* atrial fibrillation, *ACE-I/ARB* angiotensin converting enzyme inhibitors/angiotensin II receptor blocker, *cCa* corrected calcium

### Association of BNP levels, NT-proBNP levels, and the NT-proBNP/BNP ratio with non-fatal CVD events, CVD events, and MACE in CKD stages 4 and 5

**Fig. 3:**
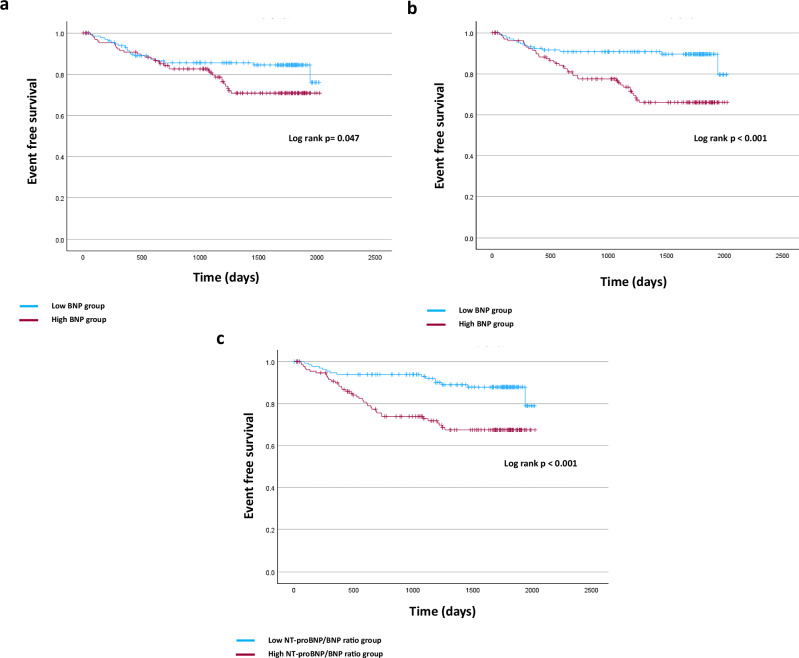
Rates of each outcome among patients with CKD stages 4 and 5 according to BNP levels. **a** Non-fatal cardiac events. **b** All cardiac events. **c** MACE. CKD chronic kidney disease, BNP brain natriuretic peptide, MACE major adverse cardiovascular events

**Fig. 4:**
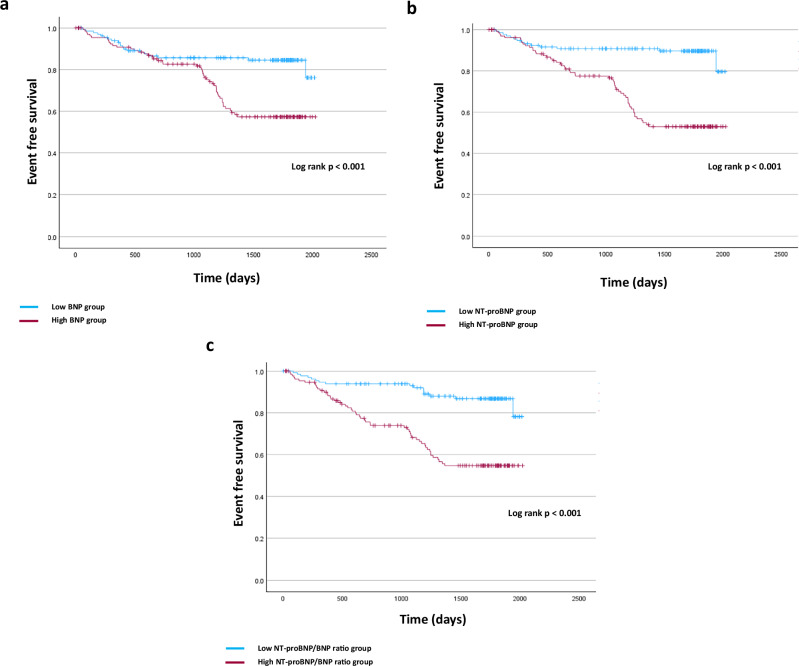
Rates of each outcome among patients with CKD stages 4 and 5 according to NT-proBNP levels. **a** Non-fatal cardiac events. **b** All cardiac events. **c** MACE. CKD chronic kidney disease, NT-proBNP N-terminal pro-brain natriuretic peptide, MACE major adverse cardiovascular events

**Fig. 5:**
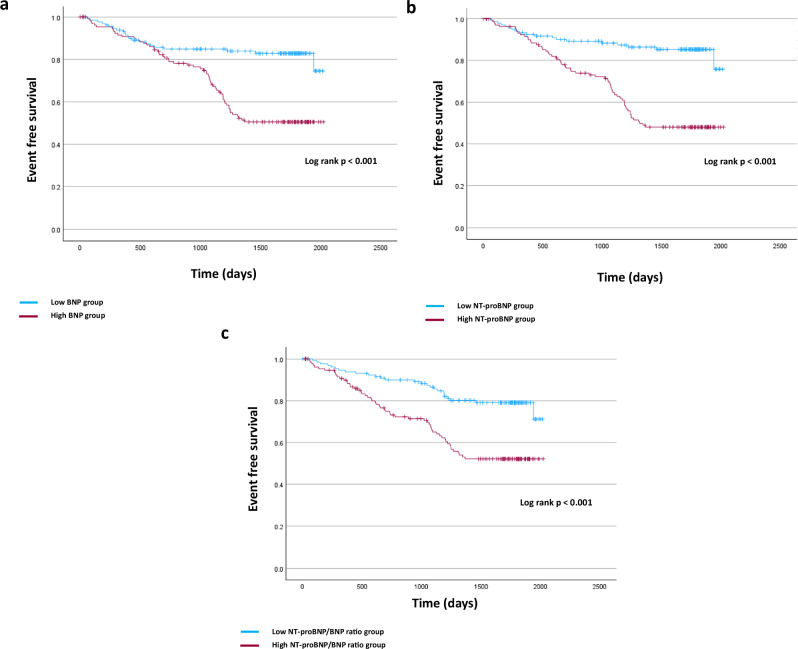
Rates of each outcome among patients with CKD stages 4 and 5 according to the NT-proBNP/BNP ratio. **a** Non-fatal cardiac events. **b** All cardiac events. **c** MACE. CKD chronic kidney disease, BNP brain natriuretic peptide, NT-proBNP N-terminal pro-brain natriuretic peptide, MACE major adverse cardiovascular events

Patients with higher BNP and higher NT-proBNP levels and higher NT-proBNP/BNP ratio had a higher prevalence of non-fatal CVD, CVD events, and MACE compared with those with lower values. Figures 3–5 show the Kaplan–Meier curves for each event depending on the BNP levels, NT-proBNP levels, and the NT-proBNP/BNP ratio. In the crude analyses, the BNP level was a predictor of non-fatal CVD events, the NT-proBNP level was a predictor of CVD events and MACE, and the NT-proBNP/BNP ratio was a predictor of all clinical outcomes. After adjusting confounding clinical factors, Cox regression analysis showed that only a high NT-proBNP/BNP ratio was associated with a significant increase in all these clinical outcomes (non-fatal CVD, hazard ratio [HR]: 1.065, 95% confidence interval [CI]: 1.019–1.112, *p* = 0.005; CVD, hazard ratio [HR]: 1.062, 95% confidence interval [CI]: 1.018–1.108, *p* = 0.005; MACE, hazard ratio [HR]: 1.054, 95% confidence interval [CI]: 1.017–1.092, *p* = 0.005) (Table 6).

**Table 6:** Hazard ratio of each clinical event by each clinical parameter in patients with CKD stages 4 and 5

	CVD				Non-fatal CVD				MACE			
	Unadjusted		Adjusted		Unadjusted		Adjusted		Unadjusted		Adjusted	
	HR (95%CI)	*P*	HR (95%CI)	*P*	HR (95%CI)	*P*	HR (95%CI)	*P*	HR (95%CI)	*P*	HR (95%CI)	*P*
BNP	1.001 (1.000–1.001)	0.012	1.000 (0.999–1.002)	0.863	1.000 (0.999–1.001)	0.853	1.000 (0.998–1.002)	0.979	1.000 (1.000–1.001)	0.052	1.000 (0.999–1.001)	0.769
NT-proBNP	1.000 (1.000–1.000)	0.001	1.000 (1.000–1.000)	0.967	1.000 (1.000–1.000)	0.650	1.000 (1.000–1.000)	0.620	1.000 (1.000–1.000)	0.011	1.000 (1.000–1.000)	0.980
NT-proBNP/BNP	1.059 (1.033–1.086)	<0.001	1.062 (1.018–1.108)	0.005	1.055 (1.026–1.084)	<0.001	1.065 (1.019–1.112)	0.005	1.049 (1.024–1.075)	<0.001	1.054 (1.017–1.092)	0.004
Hb	0.829 (0.680–1.011)	0.064	0.974 (0.744–1.275)	0.847	0.865 (0.700–1.067)	0.865	0.982 (0.737–1.307)	0.900	0.832 (0.698–0.993)	0.042	0.919 (0.743–1.136)	0.434
eGFR	0.821 (0.766–0.880)	<0.001	0.816 (0.740–0.901)	<0.001	0.837 (0.779–0.899)	<0.001	0.808 (0.727–0.899)	<.001	0.913 (0.869–0.959)	<0.001	0.911 (0.848–0.979)	0.011
P	2.240 (1.726–2.906)	<0.001	1.281 (0.845–1.940)	0.243	1.987 (1.490–2.650)	<0.001	1.229 (0.783–1.929)	0.370	1.724 (1.344–2.212)	<0.001	1.237 (0.851–1.799)	0.266
int-PTH	1.000 (0.999–1.001)	0.966	0.997 (0.996–0.999)	<0.001	1.000 (0.998–1.001)	0.757	0.997 (0.995–0.999)	<.001	0.999 (0.998–1.001)	0.419	0.998 (0.996–0.999)	0.002
U-Pro	1.049 (0.974–1.129)	0.203	0.989 (0.897–1.089)	0.818	0.938 (0.838–1.050)	0.263	0.882 (0.769–1.012)	0.073	1.010 (0.938–1.087)	0.796	0.968 (0.889–1.055)	0.457

Adjusted by Hb, eGFR, P, i-PTH, U-Pro

*CKD* chronic kidney disease, *CVD* cardiovascular disease, *MACE* major adverse cardiovascular events, *BNP* brain natriuretic peptide, *NT-proBNP* N-terminal pro-brain natriuretic peptide, *Hb* hemoglobin, *eGFR* estimated glomerular filtration rate, *P* phosphate, *i-PTH* intact parathyroid hormone, *U-Pro* urinary protein

## Discussion

This study revealed that the BNP level, NT-proBNP level, and NT-proBNP/BNP ratio increased as kidney function declined, and that changes in NT-proBNP levels were remarkable especially in CKD stage 4. In addition, interestingly, hemoglobin, phosphate, and i-PTH levels were significantly related to BNP levels, NT-proBNP levels, and the NT-proBNP/BNP ratio in patients with CKD and not on dialysis. Moreover, Cox regression analysis showed that only a high NT-proBNP/BNP ratio was associated with a significant increase in the incidence of non-fatal CVD, CVD, and MACE in patients with CKD stages 4 and 5.

In patients with CKD, the interpretation of biomarkers, such as BNP and NT-proBNP, is challenging as an impaired kidney function also contributes to their elevation. Recently, the use of ARNI has increased in patients with HF and severe HT, resulting in more frequent assessments of NT-proBNP levels as ARNI can raise BNP levels. However, as shown in our results, it has been reported that both BNP and NT-proBNP are influenced by kidney function [10, 12–15], and NT-proBNP is particularly susceptible to the effects of renal function, with significant increases observed from CKD stage 4. Furthermore, various other factors are believed to contribute to increase in BNP and NT-proBNP levels in patients with CKD.

In a previous study wherein NT-proBNP was evaluated in 209 patients without HF or CKD, NT-proBNP levels were significantly higher in those with anemia, and a significant negative correlation between levels of NT-proBNP and hemoglobin was observed [16]. Patients with CKD commonly develop anemia due to renal anemia associated with declining kidney function. In our study, hemoglobin levels were a significantly correlated with BNP levels, NT-proBNP levels, and the NT-proBNP/BNP ratio even after adjusting for various factors. The concept of cardiorenal anemia syndrome highlights that many patients with HF also have anemia [17]. Therefore, it should be considered that NT-proBNP levels may be elevated not only due to cardiac factors but also due to anemia.

Interestingly, in our study, serum phosphate and PTH levels were significantly correlated with not only BNP and NT-proBNP levels but also with the NT-proBNP/BNP ratio even after adjusting for various factors. Serum phosphate and PTH were reportedly significantly correlated with serum NT-proBNP levels [18]. As CKD progresses, abnormalities in bone mineral metabolism, known as CKD-mineral bone disorder (CKD-MBD), become obvious. These abnormalities are related to the progression of vascular calcification and development of CVD [19]. Elevated phosphate and PTH levels lead to an increase in fibroblast growth factor 23 (FGF23), which is a phosphaturic hormone. FGF23 plays a significant role in the development of CVD, specifically in HF; increased BNP and NT-proBNP levels may reflect this connection. Our results indicate that appropriate management of CKD-MBD may be essential for preventing CVD development; conversely, when BNP and NT-proBNP levels are elevated, it may be necessary to evaluate whether CKD-MBD is adequately managed.

Furthermore, as mentioned above, BNP and NT-proBNP levels increase with declining kidney function, complicating their interpretation. The NT-proBNP level is reportedly useful for predicting CVD events and mortality even in cases of impaired renal function [20–22]. However, assessing NT-proBNP levels in individuals with renal dysfunction is challenging, and only the BNP level is reportedly more closely associated with cardiac dysfunction [23]. Furthermore, in cases of kidney dysfunction, the cutoff NT-proBNP levels are elevated, suggesting that these reference values should be adjusted based on kidney function [24, 25]. Previous studies include only a few patients with CKD stages 4 and 5, which is when NT-proBNP levels begin to rise sharply, leaving evaluations in this population insufficient. Therefore, our study investigated the entire spectrum of CKD and included patients with CKD stages 4 and 5 and those on dialysis. Among those with CKD stages 4 and 5, patients with high BNP and NT-proBNP levels and high NT-proBNP/BNP ratio showed significantly higher rates of non-fatal CVD, all CVD, and MACE compared with those with low levels. However, in the multivariate analysis, only the NT-proBNP/BNP ratio remained significantly associated with these events. Notably, this study demonstrated that decreases in hemoglobin levels and CKD-MBD are related to these elevations, indicating the potential influence of various CVD risk factors. Consequently, as interpreting BNP and NT-proBNP levels is challenging particularly in patients with advanced CKD stages, the NT-proBNP/BNP ratio may be more useful as a risk predictor for CVD development. There are several possible mechanisms for the elevation of the NT-proBNP/BNP ratio. Previous studies have reported that hypoalbuminemia is associated with elevated NT-proBNP levels [26], and that inflammatory cytokines can increase both BNP and NT-proBNP concentrations [27]. It is also known that BNP and NT-proBNP differ in their metabolic and excretory pathways, as well as in their half-lives [12]. In cases of impaired renal function, discrepancies between these concentrations are more likely to occur. In fact, some reports have shown that NT-proBNP/BNP is associated with CRP, nutritional status, and eGFR [13]. Therefore, especially in advanced stages of CKD, the NT-proBNP/BNP ratio may increase in conditions characterized by high inflammatory activity or increased cardiac stress leading to enhanced BNP production.

This study has several limitations. First, it was conducted at a single institution and relied on a retrospective observational design. Therefore, the findings may not be generalizable to broader populations or different healthcare settings. Additionally, potential biases inherent in retrospective studies, such as unmeasured confounders, may affect the findings. Second, although multivariate analysis was employed, residual confounding factors may remain, particularly treatments for anemia and hyperphosphatemia; these may have influenced the BNP and NT-proBNP levels. Third, as the observation period preceded the availability of ARNI in clinical practice, the results do not account for the potential effects of ARNI. The effects of ARNI on BNP and NT-proBNP levels were not evaluated in this study, which could influence their interpretation. Fourth, the study focused on cross-sectional measurements of BNP, NT-proBNP, and their ratio without evaluating longitudinal changes or trends, which may provide additional insights into their prognostic utility.

### Perspective of Asia

In Asia, CKD is highly prevalent, and the clinical evaluation of CVD risk in these patients remains a major challenge. This study, conducted in a Japanese cohort, highlights the complexity of interpreting natriuretic peptides—BNP and NT-proBNP—in patients with CKD, which may differ from Western populations due to differences in etiology, healthcare practices, and the accessibility of dialysis therapy.

In Asian clinical settings, where NT-proBNP is increasingly used due to the adoption of ARNI, understanding the influence of CKD stage on biomarker levels is particularly critical. The present study demonstrates that the NT-proBNP/BNP ratio may serve as a more reliable prognostic indicator for CVD than either biomarker alone in advanced CKD. This may has direct implications for refining diagnostic criteria and cardiovascular risk stratification tailored to the CKD population.

Furthermore, the association of natriuretic peptide levels with anemia and CKD-MBD, which are highly relevant in CKD management, underscores the need for region-specific strategies in interpreting these biomarkers. These findings may inform updates to regional clinical guidelines, promoting more precise and culturally contextualized risk assessment.

## Conclusion

Plasma BNP and NT-proBNP levels are influenced by several factors in patients with CKD, necessitating careful interpretation. In patients with advanced-stage CKD, the NT-proBNP/BNP ratio may be a useful predictor of CVD development.

## Supplementary information

Supplemental Table 1

Supplemental Figure 1

Supplemental Figure 1 legend
