# Supplementary material for: Comparative analysis of plasma BNP and NT-proBNP levels, and NT-proBNP/BNP ratio in patients with chronic kidney disease
Source: Hypertens Res. 2025 Jul 1;48(9):2303–14. doi: 10.1038/s41440-025-02272-2 (PMC12411222; doi:10.1038/s41440-025-02272-2)
Supplement: Supplementary file 1 — Supplemental Table 1 [file 41440_2025_2272_MOESM1_ESM.docx]

Supplementary Table 1. Patients characteristics by NT-proBNP/BNP ratio group

|  | NT-proBNP/BNP ratio | | |
| --- | --- | --- | --- |
|  | <5.36  N=347 | 5.36-<10.61  N=344 | ≥10.61  N=345 |
| Age (year) | 61.8±16.9 | 63.2±17.3 | 63.3±14.8 |
| Male (%) | 220 (63.4) | 228 (66.3) | 210 (60.9) |
| Smoking (%) | 172 (49.6) | 178 (51.7) | 181 (52.5) |
| HT (%) | 280 (80.7) | 305 (88.7) | 315 (91.3) |
| DM (%) | 112 (32.3) | 98 (28.5) | 119 (34.5) |
| HLp (%) | 185 (53.3) | 179 (52.0) | 202 (58.6) |
| CHF (%) | 27 (7.8) | 29 (8.4) | 39 (11.3) |
| CAD (%) | 44 (12.7) | 42 (12.2) | 57 (16.5) |
| AF (%) | 21 (6.1) | 32 (9.3) | 28 (8.1) |
| ACE-I/ARB (%) | 223 (64.3) | 204 (59.3) | 197 (57.1) |
| Β-blocker (%) | 101 (29.1) | 119 (34.6) | 117 (33.9) |
| Statin (%) | 158 (45.5) | 162 (47.1) | 184 (53.3) |
| Hb (g/dL) | 12.5±1.9 | 11.2±1.6 | 10.1±1.6 |
| Alb (g/dL) | 3.9±0.6 | 3.8±0.7 | 3.5±0.7 |
| BUN (mg/dL) ^*^ | 27.3±17.6 | 40.4±19.4 | 60.4±23.1 |
| Cre (mg/dL) ^*^ | 1.24  (0.88-1.86) | 2.22  (1.61-3.51) | 5.60  (3.40-8.26) |
| eGFR  (mL/min/1.73m^2^) ^*^ | 46.8±24.6 | 26.7±16.9 | 13.2±10.5 |
| CKD stage |  |  |  |
| CKD 1-2 (%) | 96 (27.7) | 17 (4.9) | 1 (0.3) |
| CKD 3 (%) | 148 (42.7) | 95 (27.6) | 13 (3.8) |
| CKD 4 (%) | 79 (22.8) | 129 (37.5) | 58 (16.8) |
| CKD 5 (%) | 21 (6.1) | 94 (27.3) | 183 (53.4) |
| CKD 5D (%) | 3 (0.9) | 9 (2.6) | 90 (26.1) |
| cCa (mg/dL) | 9.4±0.7 | 9.3±0.8 | 9.0±0.9 |
| P (mg/dL) | 3.5±0.8 | 3.7±0.8 | 4.6±1.3 |
| CRP (mg/dL) | 0.09  (0.04-0.25) | 0.16  (0.06-0.64) | 0.35  (0.08-1.57) |
| U-Pro (g・gCr) ^*^ | 0.38  (0.12-1.60) | 0.83  (0.22-2.60) | 2.13  (0.77-4.83) |
| i-PTH (pg/mL) | 71.2  (47.5-111.9) | 110.9  (67.5-176.0) | 217.0  (112.5-367.2) |

CKD, chronic kidney disease; HT, hypertension; DM, diabetes mellitus; HLp, hyperlipidemia; CHF, chronic heart failure; CAD, coronary artery disease; AF, atrial fibrillation; ACE-I/ARB, angiotensin converting enzyme inhibitors/angiotensin II receptor blocker; Hb, hemoglobin; Alb, albumin; Cre, creatinine; eGFR, estimated glomerular filtration rate; cCa, corrected calcium; P, phosphate; CRP, C-reactive protein; U-Pro, urinary protein; i-PTH, intact parathyroid hormone; hs-Trop T, high sensitivity troponin T.

^*^: These are mean values in patients with CKD stage 1 to 5.
