# Supplementary figures and images for: Comparative analysis of plasma BNP and NT-proBNP levels, and NT-proBNP/BNP ratio in patients with chronic kidney disease
Source: Hypertens Res. 2025 Jul 1;48(9):2303–14. doi: 10.1038/s41440-025-02272-2 (PMC12411222; doi:10.1038/s41440-025-02272-2)

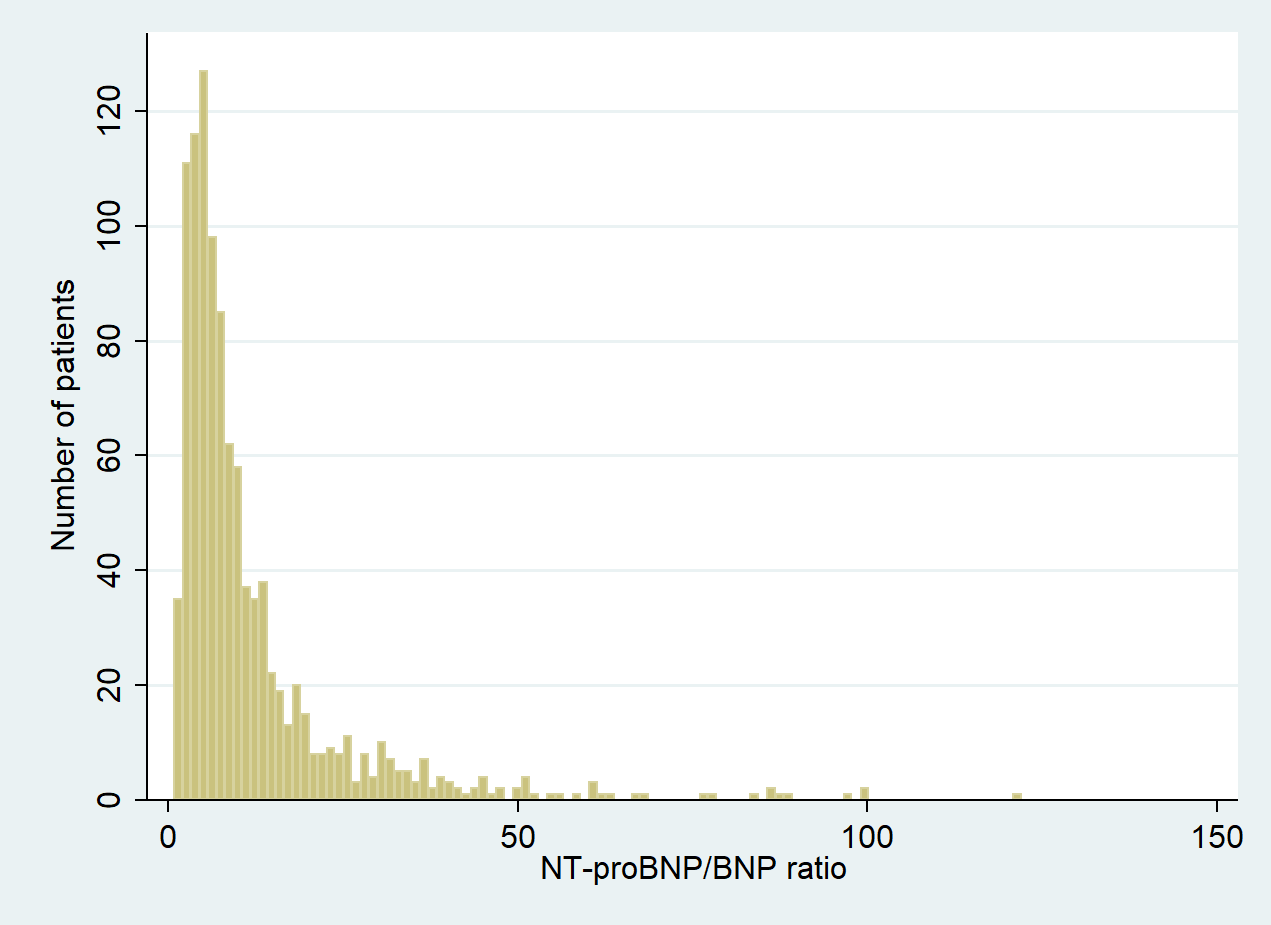

Supplement: Supplementary file 2 — Supplemental Figure 1 [file 41440_2025_2272_MOESM2_ESM.tif]
