# Supplementary material for: Comparative analysis of plasma BNP and NT-proBNP levels, and NT-proBNP/BNP ratio in patients with chronic kidney disease
Source: Hypertens Res. 2025 Jul 1;48(9):2303–14. doi: 10.1038/s41440-025-02272-2 (PMC12411222; doi:10.1038/s41440-025-02272-2)
Supplement: Supplementary file 3 — Supplemental Figure 1 legend [file 41440_2025_2272_MOESM3_ESM.docx]

**Supplementary Figure 1. Distribution of the BNP/NT-proBNP ratio among study patients**

BNP, brain natriuretic peptide; NT-proBNP, N-terminal pro-brain natriuretic peptide.
